# Atypically Displaced Meniscal Tears: An Educational Review with Focus on MRI and Arthroscopy

**DOI:** 10.3390/clinpract15060109

**Published:** 2025-06-12

**Authors:** Paolo Spinnato, Paola Franceschi, Giuseppe Martinese, Anna Parmeggiani, Valerio D’Agostino, Silvia Ferraro, George R. Matcuk, Stefano Zaffagnini, Alberto Grassi

**Affiliations:** 1 Diagnostic and Interventional Radiology, IRCCS Istituto Ortopedico Rizzoli, 40136 Bologna, Italy; 2 Department of Imaging, Cedars-Sinai Medical Center, Los Angeles, CA 90048, USA; 3 2nd Orthopaedic and Traumatologic Clinic, IRCCS Istituto Ortopedico Rizzoli, 40136 Bologna, Italy

**Keywords:** arthroscopy, diagnostic imaging, knee, magnetic resonance imaging, menisci, tears

## Abstract

This review article on atypically displaced meniscal tears serves as a critical reminder for radiologists and orthopedic surgeons. It highlights and details uncommon lesions that may be overlooked during MRI evaluation and/or arthroscopic exploration. The knowledge of their existence can enable radiologists to critically assess any meniscal abnormality, keeping in mind its possible arthroscopic presentation. This is essential for assisting the surgeon in making an accurate preoperative diagnosis. In fact, these atypical lesions pose great challenges to surgeons in terms of the technical aspects of their treatment. Often, they could require additional arthroscopic portals for their identification or the need for special devices or instrumentations for the repair. Knowing these challenges in advance is thus imperative for properly planning a proficient surgery. The correct diagnosis and description of tear patterns, including extent and location, allow optimal pre-operative planning with the choice of the indicated approach. Radiologists should know how to recognize menisci tears, even with atypical dislocation patterns. Particularly, in the case of ‘minus’ detection or thickness reduction in a meniscus, the possible displaced fragment should be carefully searched for, even in atypical sites.

## 1. Introduction

**Figure 1:**
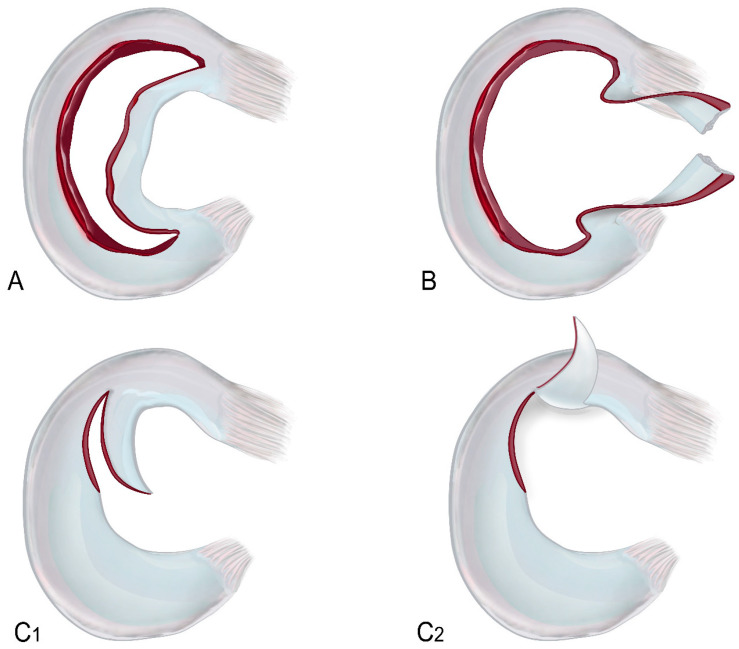
Graphical representation of the most common types of meniscal displacement. (**A**) Bucket-handle lesion; (**B**) intercondylar fragments; (**C1**,**C2**) horizontal and vertical flaps.

In general, meniscal lesions show up as (i) displaced meniscal tears that may stay attached to the parent meniscus (flap) or separate from it (fragment); (ii) substance loss, in which the meniscus loses its typical triangular shape; (iii) and simple tears, which are defined by an aberrant intrameniscal signal that extends to the meniscus surface [1,2]. The distribution of location and orientation of these tears can vary. In a recent study conducted on an adult population, horizontal tears were the most common (27%), followed by oblique (19%), radial (18%), bucket-handle (16%), longitudinal (12%), and complex tears (8%) [3]. Another study focused on 880 pediatric patients showed a higher prevalence of vertical tears (31%) [4]. Meniscal tears typically present with knee pain, swelling, locking, and instability [3,5]. The severity and type of symptoms depend on the tear’s location and pattern. For example, bucket-handle tears present with severe locking, pain, and inability to fully extend the knee [6]. Some cases may present unusually, such as a painless lump (mimicking a soft-tissue tumor) [7] or chronic knee pain without trauma, typically in chronic myxoid degenerative disease [8]. Displaced meniscal tears are a subset of meniscal lesions and are often clinically relevant and unstable. These types of tears may present with a displaced flap still attached to the torn meniscus, or a fragment subluxated at a distance from it [1]. Reattachment or resection are effective treatments for these tears. They include flap tears, bucket-handle tears, and free fragment displacements. Among these, bucket-handle tears are by far the most common ones. The most common types of displaced meniscal tears are summarized in Figure 1, with particular regard to the locations of the most common displaced fragments.

Displaced meniscal tears, except free fragment displacements, are unstable tears characterized by a flap still attached to the torn meniscus that tends to be subluxated at a variable distance from the parent meniscus. Typically, these displaced flaps are clinically significant because of the pain (up to 66.7% in posterior root tears) [9] and mechanical symptoms (such as locking, catching, and joint instability) they can cause. It is important to identify them because they can be treated successfully with reattachment (repair) or resection (partial meniscectomy) [1].

The aim of our review article is to highlight the importance of the correct diagnosis in atypically displaced meniscal tears, providing several illustrative cases with MRI and arthroscopy correlation. An overview of the basic interpretation of meniscus tears, as well as a discussion with relevant data from the literature, is provided. The final goal of this article is to improve the management of these patients, disseminating the knowledge in this field to the medical community. The current article is of particular interest to musculoskeletal radiologists, orthopedic surgeons, physiatrists, and other physicians involved in the care of these patients.

## 2. Basic Interpretation of Meniscus Tear

### 2.1. Imaging of Menisci

The imaging evaluation of knee menisci is classically provided by Magnetic Resonance Imaging (MRI). Indeed, MRI provides a comprehensive and accurate evaluation of all the parts of the menisci, including surrounding structures, intra- and extra-articular, that can contribute to the complex alterations causing the patients’ symptoms (e.g., bone, cartilage, ligaments, synovial structures, tendons, and muscles) [10]. While the role of conventional radiography is mainly limited to the exclusion of bone pathologies, calcifications, and indirect evaluation of joint structures by the analysis of articular joint space width, ultrasound may offer interesting and valuable information [11,12]. Diagnostic ultrasound can be used as a screening technique and can accurately show a range of meniscal diseases. Several studies demonstrated that ultrasound performed by experienced professionals offers acceptable sensitivity (77.5% to 88.8%) and specificity (83.8% to 84.6%) in detecting meniscal tears [13]. Other advantages of ultrasound imaging are the possibility of easily performing dynamic and weight-bearing studies that may help in understanding meniscal pathologies [14,15].

CT arthrography can be useful for meniscal evaluation, particularly when there is a contraindication to MRI [16]. Moreover, MR arthrography can be useful in the evaluation of patients with prior meniscal surgeries to evaluate for re-tear.

### 2.2. MRI and Meniscal Tears

MRI is considered the gold standard imaging technique to assess the knee menisci [17]. MRI is reliable in the detection of meniscal tears and identification of meniscal fragmentation and displacement, playing a fundamental role in surgical planning. Nevertheless, these fragments can be overlooked both at MRI and arthroscopy, especially for atypically displaced tears, increasing the risk of missed diagnosis or fragment retention after surgical treatment and leading to poor outcomes with the persistence of symptoms [2,3]. To aid in their detection, the common locations have been described both on MRI and arthroscopy, and the typical displacement patterns have been defined [2,18,19,20,21,22,23,24].

Lateral meniscal tears are more frequently overlooked compared to medial ones [23]. Meniscal fragment detection can be enhanced by identifying meniscal lesion indicators secondary to migration, particularly those with mechanical impingement and epiphyseal irritation. Historically, identifying radial and peripheral tears, as well as those near the posterior horn insertion, has been challenging. However, advancements in arthroscopic expertise, improved recognition of common lesion patterns, and the ability to select the optimal MRI sequence and imaging plane for each lesion type have significantly enhanced diagnostic accuracy. When used appropriately, MRI remains an essential tool for detecting all types of meniscal tears [25,26].

Flap tears occur six to seven times more frequently in the medial meniscus, where in two-thirds of cases, fragments are displaced posteriorly (near or posterior to the posterior cruciate ligament (PCL). In the remaining cases, flaps displace into either the intercondylar notch or in the medial meniscal gutter with flaps lying in the superior (meniscofemoral) recess more often than in the inferior (meniscotibial) recess. Lesions of the lateral meniscus are less common and generate displaced flaps equally distributed along the posterior joint line and lateral recess. Meniscal flaps may also be displaced into newly formed cavities or fissures that are not physiologically present, as in the case of meniscal entrapment in a tibial plateau fracture or tibial spine avulsion [15]. The identification of the displaced flap is crucial for optimal surgical planning. Finally, the whole meniscus can be displaced from its native position in the cases of root tears. In fact, the interruption of the fixation from the tibia at the level of the posterior or anterior root may cause the meniscal body to extrude beyond the tibial plateau.

#### MRI Reporting Recommendations

It is preferable to characterize the site of meniscal pathology as anterior horn, body, or posterior horn, in conjunction with circumferential inner, mid, or outer sections [10]. According to the European Meniscus Consensus, the pathology should be recorded as a simple meniscal “lesion” unless there has been a “sufficient” previous knee trauma in the clinical history, in which case the terms meniscal tears or ruptures should be used [10].

Furthermore, they advise against using MRIs to determine the stability of small meniscal tears, because this is an arthroscopic diagnosis. However, a big tear (distance between flaps > 5 mm), which could cause a locked knee, or a radial tear may be described as an unstable meniscal tear [10].

Eight arthroscopic findings are included in the International Society of Arthroscopy, Knee Surgery, and Orthopaedic Sports Medicine (ISAKOS) categorization [27]:

(1) Tear depth classifies tears as complete if they penetrate both the superior and inferior meniscal surfaces and partial if they penetrate only one of them.
(2) The Cooper zone categorization of meniscal tears is used to assess the rim width, or circumferential location, based on the extent to which the tear spreads centrally into the meniscus. Cooper Zone 1 (rim width < 3 mm; outer third), Cooper Zone 2 (rim width 3 mm to <5 mm; middle third), and Cooper Zone 3 (rim width ≥ 5 mm; inner third) are the three areas into which tears are classified. Evidence of vascularity up to 3 mm from the meniscus’s perimeter led to the selection of the 3 mm threshold. A tear is graded based on the outermost zone involved (if multiple zones are involved), such as a radial tear. It is not recommended to use historical “red-red,” “red-white,” or “white-white” nomenclature.
(3) The meniscus is divided into thirds by radial location topographic classification, which classifies tears according to their extension into the anterior, body, or posterior zones. Multiple zones can be torn at the same time; for example, a full bucket-handle tear would affect all three zones. Alternatively, unless a radial tear crosses the mid-body, the radial location can be classified as either anterior or posterior, splitting the meniscus in half.
(4) If lateral meniscal tears reach entirely or partially in front of the popliteal hiatus, they are rated as central to the hiatus.
(5) Tear patterns might be horizontal, radial, vertical flap, horizontal flap, longitudinal–vertical (which incorporates a bucket-handle tear as an extension), or complex. With the exception of complex tears, which include two or more tear patterns, the main tear pattern should be mentioned.
(6) Whether the meniscal tissue appears degenerative, nondegenerative, or unknown is referred to as tissue quality. Degenerative meniscal tissue is characterized by softening, fibrillations, cavitations, and/or numerous tear patterns.
(7) The length of the meniscal tear that extends into its surface is called the tear length (in millimeters, as determined using an arthroscopy ruler or probe). The measured tear length does not account for confined tears or intermeniscal degeneration.
(8) The percentage of meniscal tissue surface area that is removed arthroscopically is known as the “percentage of the meniscus excised.” This percentage can be calculated quantitatively and represented graphically by crosshatching the excised tissue on a diagram.

### 2.3. Diagnostic Arthroscopy

In current clinical practice, MRI, together with clinical examinations, is usually performed to diagnose meniscal pathology and to assess its clinical relevance. However, since MRI may produce various rates of false-positive and false-negative results, arthroscopy sometimes plays a crucial role in the diagnosis or confirmation of a meniscal tear [28]. It can be considered invasive for a diagnostic purpose and should be reserved for selected cases with contradictory MRI results and clinical symptoms.

Generally, we can affirm that MRI offers, in a non-invasive manner, a wide and comprehensive evaluation of the knee, including evaluation of bone alteration and peri-articular structures, but it can be affected by diagnostic inaccuracies in selected cases. Arthroscopy may overcome these problems, but it is invasive. Arthroscopy is also more expensive than MRI and may lead to post-surgical complications.

However, ‘needle arthroscopy,’ a less invasive direct imaging technique, has been recently used to offer direct visualization of the intra-articular space, including menisci. This imaging technique can be performed with very small needles (usually 18 Gauge) compared to standard arthroscopy tools [29]. The technological progress of this equipment should encourage its use in the future.

Linking theory about diagnosis to practice is essential for developing accurate and effective clinical skills. Indeed, theoretical knowledge provides the foundation for understanding health conditions and diagnostic criteria. However, applying this knowledge in real-world settings allows practitioners to recognize how symptoms manifest in diverse individuals and contexts. Practical cases offer concrete examples that deepen understanding, highlight nuances, and ensure more accurate diagnoses and better-informed treatment decisions. Thus, in the next section, we provide a case series from our experience.

## 3. Exemplificative Cases: MRI and Arthroscopy

The aim of this section of the manuscript is to present several peculiar and educational cases of meniscal tears with flaps displaced in atypical locations. The goal is to depict their appearance both on MRI and arthroscopy, in order to increase radiological recognition and accurate reporting, and to help the orthopedic surgeon in surgical planning.

These seven cases presented below represent original material from our Institution’s archive and were selected through the years with the experience of the authors (S.Z., P.S., and A.G.). They were chosen for their rarity and diagnostic difficulty.

### 3.1. Medial Meniscus Flap Under the Medial Collateral Ligament

A 48-year-old male experienced sudden pain in the medial region of the knee after running on a mountain terrain. Subsequently, he complained of medial sharp pain with knee flexion and a sensation of clicking with knee movements on the medial side of the knee. At clinical examination, he had positive McMurray and Apley tests, pain at palpation at the medial joint line, and a stable knee.

**Figure 2:**
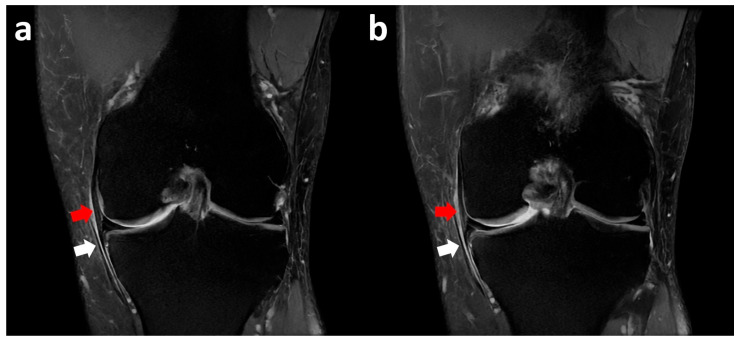
Left knee coronal fat-suppressed PD MR images show a medial meniscus body tear (**a**) with an open oblique course on the inferior side associated with a meniscal flap (white arrows) displaced inferiorly and trapped between the medial tibial bony surface and medial collateral and capsular–ligamentous complex (red arrows) (**b**).

Coronal proton density-weighted fat-suppressed (PD FS) MR images show a medial meniscus body tear with an open oblique course on the inferior side associated with a meniscal flap displaced inferiorly and trapped between the medial tibial bony surface and medial capsular–ligamentous structures that are edematous secondary to friction phenomena (Figure 2).

**Figure 3:**
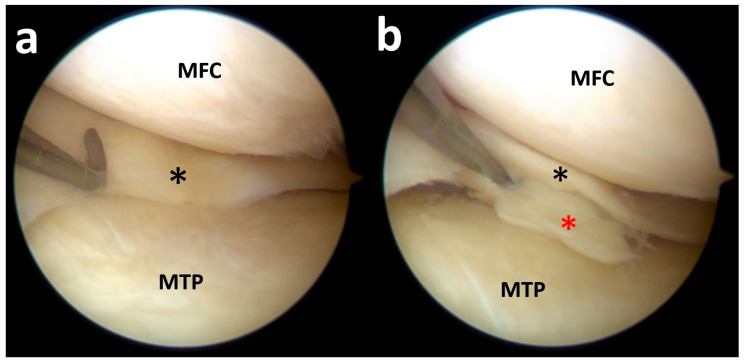
Initially, the medial meniscus body (black asterisk) did not present as a visible lesion (**a**). However, after probing the inferior surface, it was possible to find a displaced flap tear (red asterisk) amenable to partial resection (**b**). MFC, medial femoral condyle; MTP, medial tibial plateau.

The patient was treated with arthroscopic resection of the medial meniscus flap, with resolution of symptoms (Figure 3).

The initial MRI report did not correctly describe this meniscal tear, particularly the position of the flap was not reported. A successive re-evaluation by an experienced musculoskeletal radiologist clarified the type of the lesion.

### 3.2. Medial Meniscus Radial Tear with Flipped Body

A 21-year-old male professional dancer experienced sudden pain after a hyperflexion and external rotation movement while dancing. After that episode, the patient complained of medial knee pain and episodes of locking. At clinical examination, he had a full range of motion (ROM), a stable knee, medial pain, and a positive Appley test.

**Figure 4:**
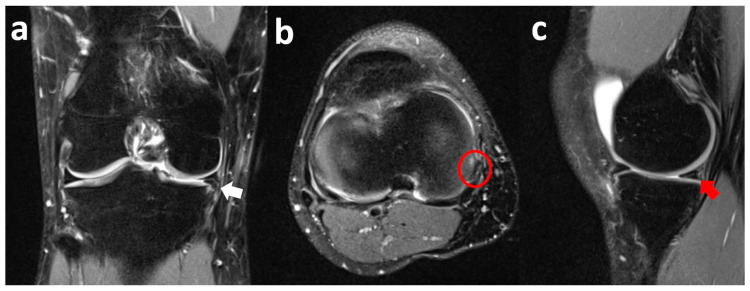
Right knee coronal fat-suppressed PD MR images (**a**) show a medial meniscus body tear with an open oblique course on the inferior side associated with a meniscal flap (white arrow) displaced inferiorly and trapped between the medial tibial bony surface and medial collateral and capsular–ligamentous complex. Axial image (**b**) shows the fragment at the level of the posteromedial tibial plateau (red circle). Sagittal images (**c**) show a normal appearance of the posterior horn (red arrow) with just a minimal defect at its free margin and a slightly hyperintense intrameniscal line.

Coronal, axial, and sagittal fat-suppressed PD MR images (Figure 4) show a medial meniscus body tear with an open oblique course on the inferior side associated with a meniscal flap displaced inferiorly; however, only a minor defect at the level of the posterior horn can be noted.

**Figure 5:**
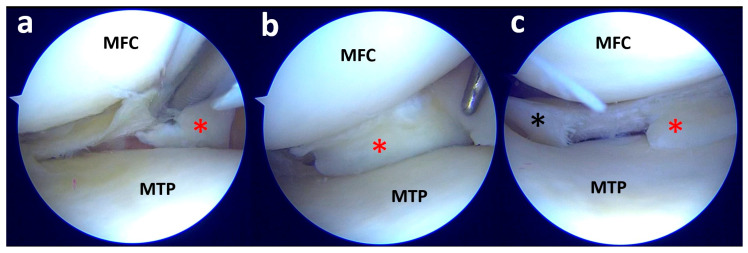
By lifting the medial meniscus body, it is possible to note a small displaced flap (red asterisk) below the medial tibial plateau (**a**); with the probe, the flap is mobilized, revealing a full extent large flap of the meniscal body and posterior horn (red asterisk) (**b**); moving the scope toward the posterior horn it is possible to note a radial tear close to the posteromedial root (black asterisk) (**c**). MTP, medial tibial plateau; MFC, medial femoral condyle.

Despite the MRI appearance, during arthroscopy, a big flap was displaced inferiorly and a radial tear at the posterior horn was noted; thus, the patient was successfully treated with arthroscopic medial meniscus repair after relocating the flap from behind the tibia (Figure 5).

The initial MRI report did not detect any meniscal tears. A successive re-evaluation by an experienced musculoskeletal radiologist, together with orthopedic surgeons, clarified the case.

### 3.3. Medial Meniscus Posterior Root Tear with Extrusion

An 11-year-old male patient experienced a hyperextension knee injury caused by a direct kick to the proximal tibia. After an initial period of rest to heal the acute trauma, he complained of medial knee pain with activities and anterior pain with knee flexion. At clinical examination, he had positive posterior drawer (grade 3+), McMurray, and Apley tests, and pain at palpation at the medial joint line.

**Figure 6:**
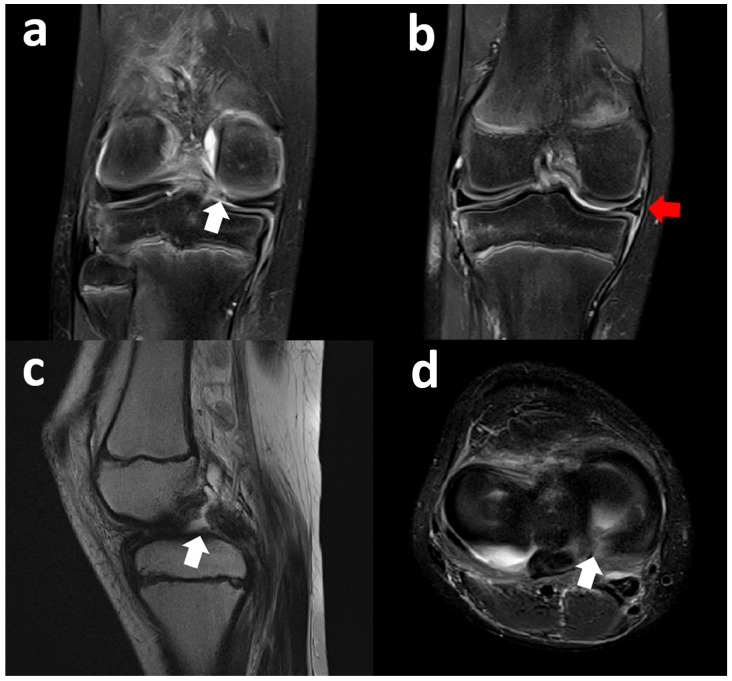
Right knee coronal fat-suppressed PD-weighted MR images show a full-thickness radial tear of the medial meniscus posterior root (white arrow) (**a**) associated with peripheral extrusion of the meniscal body (red arrow) (**b**). Sagittal T2-weighted image highlights the fibrocartilaginous defect and also shows the complete tear of the posterior cruciate ligament (white arrow) (**c**). Axial STIR sequence confirms the complete absence of the posterior root of the medial meniscus (white arrow) (**d**).

Coronal fat-suppressed PD MR images show a lesion of the medial meniscus posterior root associated with peripheral extrusion of the meniscal body. Sagittal T2-weighted and axial STIR images confirm the full-thickness radial tear of the posterior root of the medial meniscus (Figure 6).

**Figure 7:**
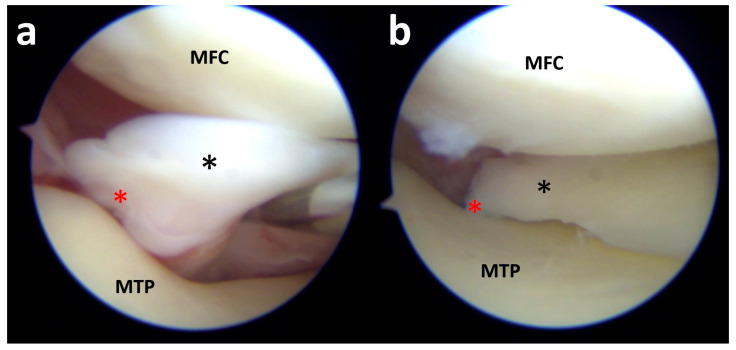
By lifting the posterior horn of the medial meniscus (black asterisk) with the probe, a detachment of the posterior root (red asterisk) from the medial tibial plateau (MTP) is found (**a**). After transosseous repair of the posterior root, the meniscus (black asterisk) becomes reinserted to the anatomical location at the tibial bone (red asterisk) (**b**). MFC, medial femoral condyle.

The patient was treated with arthroscopic physeal sparing PCL reconstruction and transosseous repair of the medial meniscus posterior root tear (Figure 7).

The initial MRI report detected the extrusion of the meniscal body without a diagnosis of the root tear. A successive re-evaluation by an experienced musculoskeletal radiologist clarified the type of the lesion.

### 3.4. Lateral Meniscus Flap in Popliteus Recess

A 32-year-old male experienced a knee injury during a motorcycle accident. He was unable to bear weight due to pain and instability. At clinical examination, he had ROM restriction (10–90°), positive 3+ Lachman, 3+ Pivot-Shift, 3+ Varus Stress test, and lateral pain.

**Figure 8:**
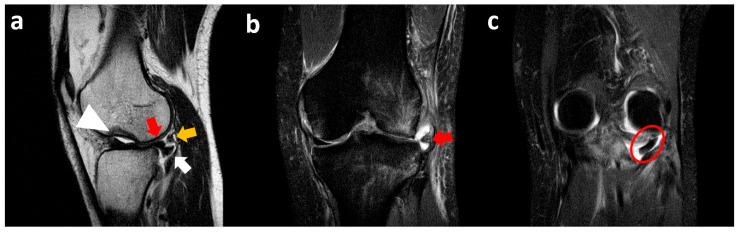
Left knee sagittal T2-weighted MR image (**a**) and coronal fat-suppressed PD MR image (**b**) show part of the lateral meniscus body (white arrow) dislocated in the popliteal hiatus below the popliteus tendon (yellow arrow) and the rest of the meniscus (red arrow). Coronal fat-suppressed PD MR image (**c**) shows lateral meniscal body extrusion (red arrow) and the inferior displacement of the meniscal portion (red circle). Bone marrow edema of the lateral tibial plateau and corresponding femoral condyle, together with an articular cortical depression (‘notch sign’—arrowhead in (**a**)), is also noted.

A sagittal T2-weighted MR image shows a meniscal flap originating from the posterior horn of the lateral meniscus, extending posteriorly into the popliteal hiatus, surrounded by joint fluid distending the popliteus recess. As a collateral finding, the “notch sign” on the lateral femoral condyle (impaction fracture associated with knee distortive trauma) is seen. Coronal fat-suppressed PD MR image confirms the presence of the meniscal flap in the medial popliteal region surrounded by articular effusion. Another coronal fat-suppressed PD MR image demonstrates concomitant lateral meniscal extrusion with abundant bone marrow edema at the level of the lateral femoral condyle and proximal tibial epiphysis (Figure 8).

**Figure 9:**
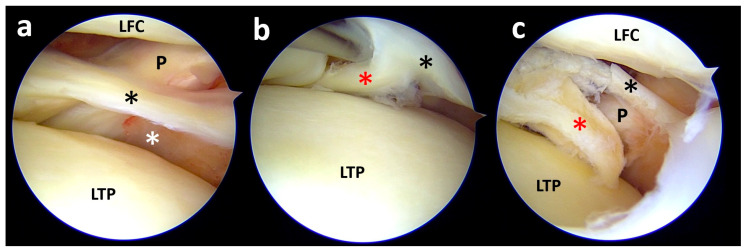
At the level of the popliteus tendon (P), it is possible to see only a thin remnant of the meniscal body (black asterisk), while most of the meniscal body is missing (white asterisk) (**a**). By lifting the meniscus remnant (black asterisk), it is possible to note the meniscal body (red asterisk), which is flipped backward and inferiorly in the interior popliteal hiatus (**b**). After reducing the displaced fragment from the popliteal hiatus (red asterisk), the lesion pattern is clear and can be approached for repair (**c**). LTP, lateral tibial plateau; LFC, lateral femoral condyle.

The patient was treated with arthroscopic anterior cruciate ligament (ACL) reconstruction plus lateral plasty, lateral collateral ligament (LCL) repair with a suture anchor, and lateral meniscus all-inside repair after mobilizing the posterior portion from the popliteal space to the anatomical location (Figure 9).

The initial MRI report did not correctly describe the meniscal tear; particularly, the position of the displaced part of the meniscus was not reported. A successive re-evaluation by the orthopedic surgeons clarified the type of lesion.

### 3.5. Lateral Meniscus Flipped in the Lateral Gutter

A 16-year-old male experienced a knee sprain during an accident while driving an all-terrain vehicle. He had knee pain during weight-bearing and knee flexion, which was limited to 100°. The patient complained sensation of instability as well. At clinical examination, he had ROM restriction (0–100°), 3+ Lachman, 3+ Pivot-Shift, and lateral pain.

**Figure 10:**
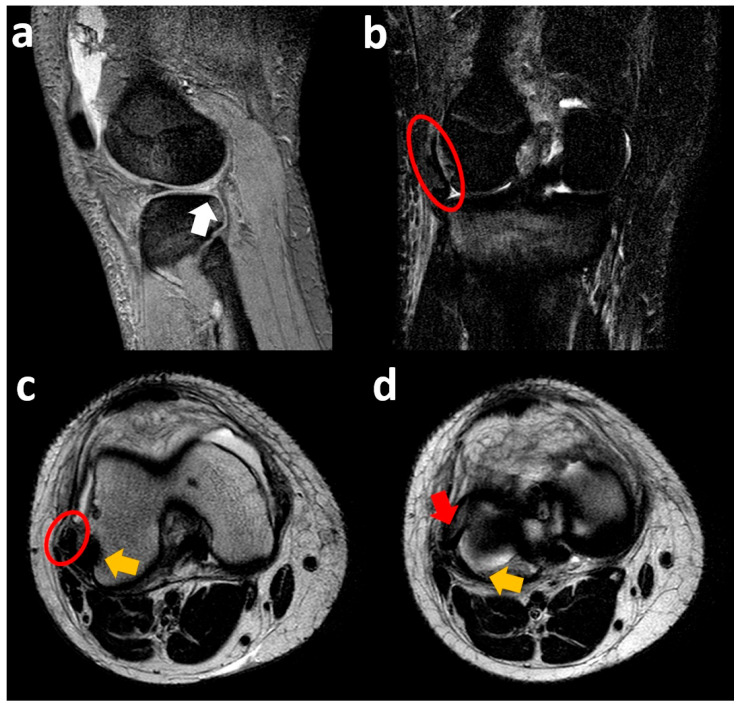
Right knee sagittal fat-suppressed T2-weighted MR image (**a**) shows a complete absence of the posterior horn of the lateral meniscus (white arrow). Coronal fat-suppressed T2-weighted MR image (**b**) shows the posterior horn meniscal flap (red circle) displaced laterally to the lateral femoral condyle. Two adjacent slices of axial T2-weighted MR images show the meniscal portion (red circle and red arrow) trapped between the popliteus tendon (yellow arrows) and the joint capsule (**c**,**d**).

The sagittal fat-suppressed T2-weighted MR image shows a complete absence of the posterior horn of the lateral meniscus concomitant with abundant suprapatellar joint effusion. Coronal fat-suppressed T2-weighted MR image shows a displaced meniscal flap from the body of the lateral meniscus arranged laterally to the lateral femoral condyle; modest bone marrow edema at the proximal tibial epiphysis is also noted. The two axial T2-weighted MR images show the meniscal flap trapped between the popliteus muscle tendon and the joint capsule (Figure 10). The patient also had a complete anterior cruciate ligament injury.

**Figure 11:**
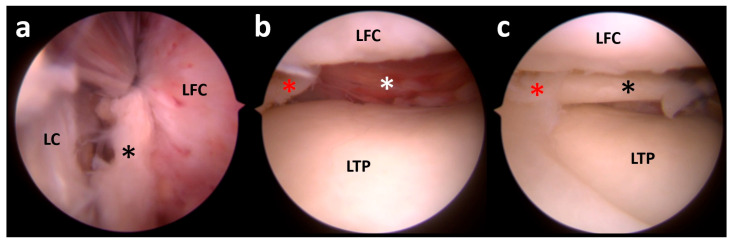
In the lateral compartment, the remnant of the meniscal body is seen (red asterisk) while the posterior horn is missing, allowing visualization of the joint capsule (white asterisk) (**a**). With the arthroscope in the lateral gutter, the lateral meniscus body and posterior horn (black asterisk) are found between the lateral capsule (LC) and the lateral femoral condyle (LFC) (**b**). After the reduction in the meniscal portion from the lateral gutter (red asterisk), the meniscal shape is restored and amenable to repair (**c**). LTP, lateral tibial plateau; LFC, lateral femoral condyle.

The patient was treated with arthroscopic ACL reconstruction plus lateral plasty; the lateral meniscus body and posterior horn were mobilized from the lateral gutter and re-positioned in their anatomical location, where a transosseous repair was performed (Figure 11).

The initial MRI report did not correctly describe this meniscal tear. A successive re-evaluation by an experienced musculoskeletal radiologist clarified the type of the lesion.

### 3.6. Lateral Meniscus Posterior Root Tear with Extrusion

A 27-year-old male experienced a knee sprain due to non-contact trauma while playing soccer. After an initial period of rest to heal the acute trauma, he complained of lateral knee pain with activities and episodes of knee instability. At clinical examination, he had full ROM, 3+ Lachman, 3+ Pivot-Shift, and lateral pain.

**Figure 12:**
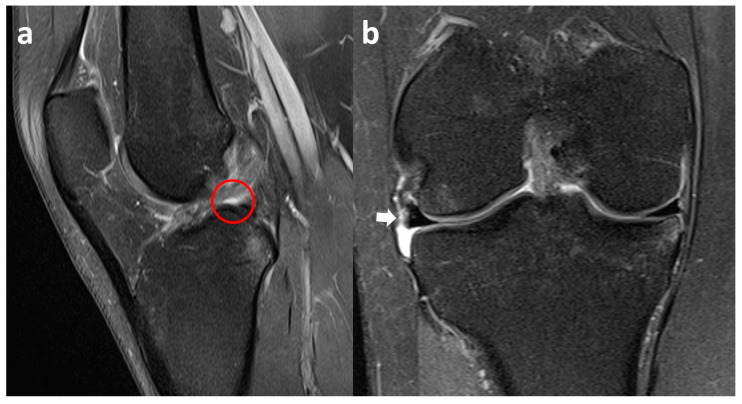
Right knee sagittal fat-suppressed PD-weighted MR demonstrates the absence of a part of the meniscal posterior horn (red circle) behind the ACL and below the PCL (**a**). Coronal fat-suppressed PD-weighted MR shows the extrusion of lateral meniscus body (white arrow) (**b**).

Sagittal and coronal fat-suppressed PD-weighted MR images are not able to identify a gross lateral meniscus lesion; however, the posterior horn is not clearly visible below the PCL, and meniscal extrusion can be appreciated (Figure 12).

**Figure 13:**
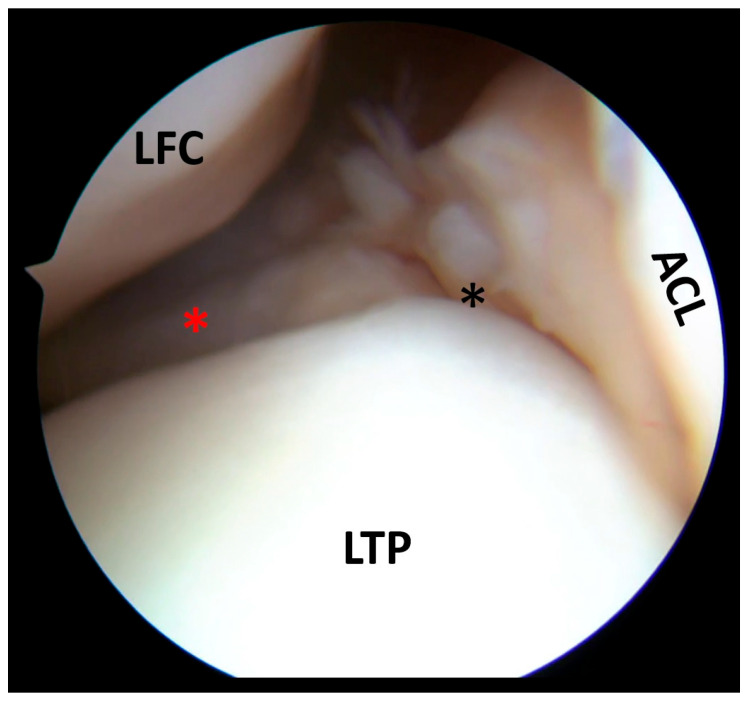
In the lateral compartment, the lateral meniscus posterior horn (red asterisk) demonstrates an interruption at the level of the posterior root (black asterisk) between the meniscus and the tibia. LTP, lateral tibial plateau; LFC, lateral femoral condyle; ACL, anterior cruciate ligament.

The patient was treated with arthroscopic ACL reconstruction plus lateral plasty and transosseous repair of the lateral meniscus posterior root tear (Figure 13).

The initial MRI report detected the extrusion of the meniscal body without a diagnosis of the root tear. A successive re-evaluation by an experienced musculoskeletal radiologist clarified the type of the lesion.

### 3.7. Lateral Meniscus Body Within Tibial Plateau Fracture

A 46-year-old male experienced a fall from height while climbing, reporting a Schatzker type II tibial plateau fracture. The patient underwent minimally invasive closed reduction and internal fixation with three cannulated screws. Six months after surgery, the fracture healed, but the patient presented with severe pain during weight-bearing and knee flexion, with knee stiffness and passive range of motion limited to 10–70°.

**Figure 14:**
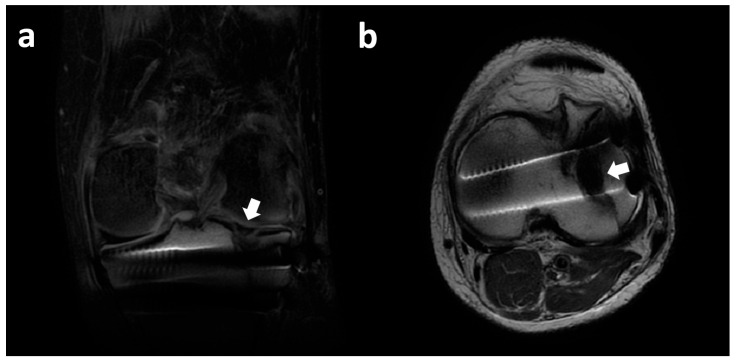
Left knee coronal fat-suppressed PD-weighted MR image (**a**) and axial T2-weighted MR image (**b**) show a portion of the lateral meniscus entrapped in the fracture rim of the tibial plateau joint face (white arrows).

The lateral tibial plateau fracture with articular involvement was fixated by metal screws. Coronal fat-suppressed PD-weighted MR image shows a portion of the lateral meniscus entrapped in the fracture rim on the tibial joint face. Axial T2-weighted MR image confirms the presence of a meniscal flap embedded in the fracture rim (Figure 14).

In this case, open surgery was preferred. More precisely, the patient was treated with hardware removal, open arthrolysis, and meniscal treatment. The lateral meniscus was released from the entrapment and sutured to the articular capsule after excision of the degenerated anterior portion.

The initial MRI report was inconclusive. A successive re-evaluation by an experienced musculoskeletal radiologist, together with orthopedic surgeons, clarified the position of the entrapped meniscus.

## 4. Discussion

In the experience of the senior surgeons (S.Z. and A.G.), these atypical meniscal tears are frequently underreported in the initial radiological assessment. In some instances, the diagnosis was only established after a secondary review or emerged as an unexpected intraoperative finding. In particular, lesions involving the medial and lateral meniscal posterior roots can pose a diagnostic challenge on MRI and are frequently overlooked [18,30,31,32,33,34,35,36,37,38,39,40]. In such cases, arthroscopic assessment and probing remain the gold standard for diagnosis, and radiologists should recommend this approach when uncertainty persists. Moreover, if the MRI performed is a low-field strength scanner, the diagnostic accuracy of these diagnoses can be even lower. Particularly, patients who underwent open MRI (usually performed with a 0.2 or 0.3 Tesla magnet) may suffer from incorrect evaluation of complex meniscal tears. A repeat MRI study with high-field equipment (1.5 Tesla or higher) is recommended in cases with uncertain clinical findings or discrepancies between the MRI results and clinical symptoms.

Lateral meniscus posterior root tears (LMPRTs), if left untreated, can lead to rapid articular cartilage degeneration and loss of the meniscus as a secondary stabilizer, significantly compromising knee function. In a retrospective study of 45 patients with arthroscopically confirmed LMPRTs (2010–2017), only 33% of tears were identified preoperatively on MRI [37]. A retrospective review revealed that 50% of missed LMPRTs were clearly visible, 40% were subtly evident, and 10% were occult and inherently undetectable. Given the high rate of missed diagnoses, MRI reports should explicitly note cases where the root is poorly visualized, particularly in patients with previous ACL reconstruction, to prompt thorough intraoperative evaluation. Surgeons should maintain a high index of suspicion and carefully assess the posterior root of the lateral meniscus during arthroscopy, especially in the context of ACL injuries.

Meniscal root tears typically arise in two contexts: chronic degeneration or acute trauma. Posterior root tears of the medial meniscus are often degenerative and non-traumatic, whereas lateral meniscus root tears are more commonly associated with acute injury [38,39].

Lateral meniscus posterior root tear contributes to anterolateral rotational instability and meniscus extrusion, often with anterior cruciate ligament injury [40,41]. Therefore, post-traumatic meniscal root repair is highly recommended for well-selected patients, as it is associated with lower rates of osteoarthritis and total knee arthroplasty compared to partial meniscectomy or nonsurgical management [42].

It is worth remembering that medial meniscus root tears are usually degenerative and associated with overweight and obese patients, frequently in women [33,34,35,36]. Meniscal extrusion is common in patients with medial meniscus root tears, while it is rare in patients with LMRTs, which are more commonly associated with a history of trauma and anterior cruciate ligament tears [43,44]. Meniscal body extrusion serves as another indirect sign when a clear “ghost sign” is not visible [33]. As a matter of fact, when the circumferential continuity of the meniscus is interrupted by a root tear, the meniscus is not able to resist the hoop stress and, thus, is prone to its peripheral displacement. Regarding patients’ age and meniscal tears, it is worth noting that the occurrence of medial meniscal tears increases with age, while isolated lateral meniscal tears are more common in individuals under the age of twenty and decline with age.

Small, displaced flaps of the medial meniscus can also represent a diagnostic challenge: They can be overlooked on MRI, if the altered shape of the meniscus is not appreciated, and can be easily missed at surgery without vigorous probing. When the flap is trapped inferiorly between the tibial plateau and the deep MCL, it is not visible during a superficial arthroscopic examination, but it should be searched by lifting the meniscal body upward and sometimes vigorously probing meniscal tissue to disengage the locked fragment, as in the cases described in Section 3.1 and Section 3.2. Another reason for optimizing the diagnosis in such cases is that if the fragment is identified and removed, the clinical improvement is almost immediate, making the arthroscopic treatment very proficient. In the opinion of the senior surgeons, an inferiorly displaced flap represents a factor for unsatisfying conservative treatment and, thus, a clear indication for surgery [45,46,47].

Displaced flaps of the lateral meniscus represent a more challenging field. Although the diagnosis and treatment of flaps displaced in the intercondylar notch are more straightforward, when the lateral meniscus occupies the popliteal recess, it could pose challenges to the surgeon, as illustrated in Section 3.4 and Section 3.5. First of all, the popliteal recess is not accessible during standard arthroscopic exploration, and special positioning or arthroscopic portals are needed; secondly, the highly vascularized area could create adhesions with the surrounding tissues, making the reduction extremely complex [48,49]. For this reason, in the presence of displaced lateral meniscal lesions, surgery becomes a priority and should be performed as soon as possible. This would maximize the chances of repairability, thus minimizing the risk of long-term osteoarthritis due to meniscal removal.

Finally, an entrapped meniscus represents an even rarer circumstance. It should be suspected in lateral tibial plateau fracture, both pre-operatively and after fixation (especially if performed minimally invasively without joint exploration). In fact, the compressive force that generated the tibial plateau fracture could detach the lateral meniscus from the periphery and push it inside the split [50,51,52]. Surgeons should be aware of this event because an entrapped meniscus could be responsible for the impossibility of fracture reduction, and, thus, joint exploration (open or arthroscopic) should be performed in these cases. Often, the meniscus seems missing or could be mistaken for a cartilage fragment and thus left in the incorrect place. This could lead to post-operative pain and poor outcomes.

Radiologists play a crucial role in detecting meniscal pathology and should be well-versed in the imaging characteristics of various meniscal tear patterns. This knowledge is essential for guiding surgical planning and optimizing patient outcomes [35].

Advancements in imaging techniques may enhance the detection of these lesions, reducing underreporting and improving subsequent treatment strategies. Deep learning algorithms have shown significant promise in knee MRI analysis, offering precise identification and localization of meniscal tears. A recent systematic review by Botnari et al. underscores the potential of deep learning models in this domain [36]. However, their clinical application remains challenging due to difficulties in accurately segmenting regions of interest (ROIs), particularly in cases involving overlapping structures or indistinct anatomical boundaries. The selection of appropriate segmentation techniques is critical to improving diagnostic accuracy. Despite these technological advances, expert musculoskeletal radiologists remain essential for verifying segmentation accuracy, which is, unfortunately, a time-consuming process that hinders the use of these technologies in daily practice [36]. Furthermore, deep learning technologies in MRI currently serve as adjuncts rather than replacements for expert musculoskeletal radiologists. Human expertise remains crucial for interpreting complex cases, integrating patient history, and making informed diagnostic decisions.

The current article has several limitations. Particularly, the literature research performed was not systematic, and the cases provided do not cover all the possible atypical displaced meniscal tears patterns, but are limited to the available authors’ institution archive. Furthermore, an inter-observer reliability analysis of the MRI interpretations was not performed and data on long-term clinical outcomes is missing.

## 5. Conclusions

In conclusion, improving the recognition and classification of atypically displaced meniscal lesions requires a combination of advanced imaging techniques, thorough radiological assessment, and arthroscopic expertise. Early and accurate diagnosis is essential for optimizing treatment outcomes and preserving knee function, particularly in cases where surgical intervention is warranted.

## Data Availability

No new data were created or analyzed in this study.
